# Isolated Intracranial Extramedullary Hematopoiesis in an Adult Patient

**DOI:** 10.5334/jbsr.3268

**Published:** 2023-08-25

**Authors:** Ramazan Orkun Onder, Alptekin Tosun, Tümay Bekci

**Affiliations:** 1 Giresun University, Faculty of Medicine, Turkey; 2 Giresun University, Faculty of Medicine, Department of Radiology, Giresun, Turkey

**Keywords:** intracranial extramedullary hematopoiesis, metastases, meningioma, chloroma, epidural hematoma

## Abstract

This article describes the case of a 56-year-old male patient who presented with a headache and swelling on the left side of his head. Medical examinations, including non-contrast computed tomography (CT) and contrast-enhanced magnetic resonance imaging (MRI), revealed the presence of an expansile soft tissue lesion in the frontoparietal bone structures, causing compression of the brain tissue. Further immunohistochemical analysis confirmed the diagnosis of extramedullary hematopoiesis (EMH) in the bone lesion, with predominant involvement of the myeloid series. EMH is a compensatory mechanism of the body to meet the demand for erythropoiesis. While EMH is commonly associated with thalassemia in children and myelofibrosis in adults, it can occur in various tissues throughout the body, including the intracranial region, although it is rare. The imaging findings of EMH on MRI typically show iso-hypointense signals on T1-weighted images and hypointense signals on T2-weighted images due to the presence of hemosiderin. However, in this case, the lesions appeared hyperintense on T2-weighted images, which posed a challenge in the differential diagnosis. The article highlights the importance of clinical evaluation and imaging findings in diagnosing EMH, as these lesions can resemble other conditions such as metastases, angiomatous meningioma, chloroma, or epidural hematoma.

**Teaching Point:**

Isolated cases of intracranial EMH should be considered as a differential diagnosis in adult patients as they may be radiologically confused with malignant conditions such as metastases, angiomatous meningioma, chloroma or epidural hematoma.

## Case History

**Figure 1:**
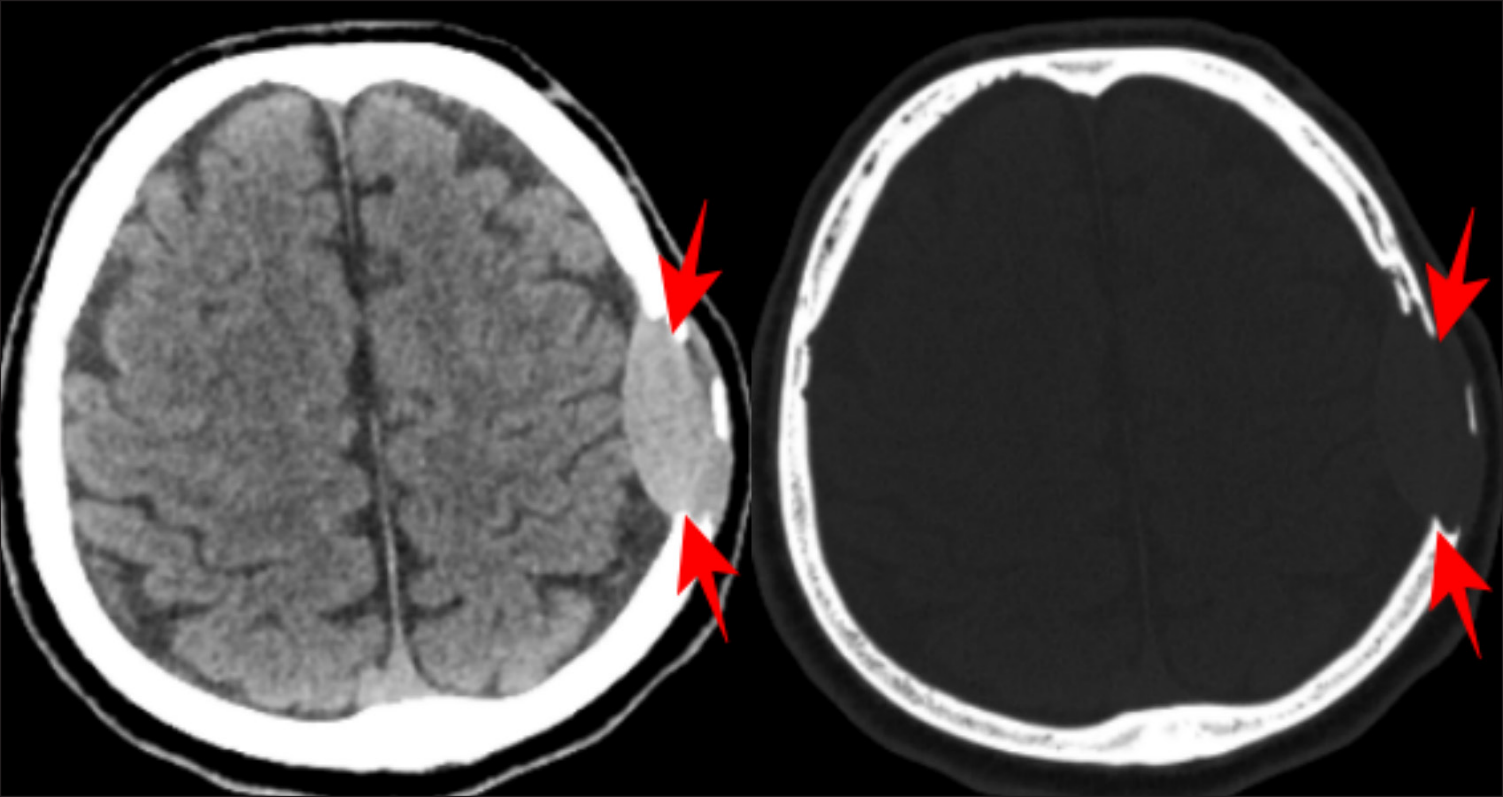


**Figure 2:**
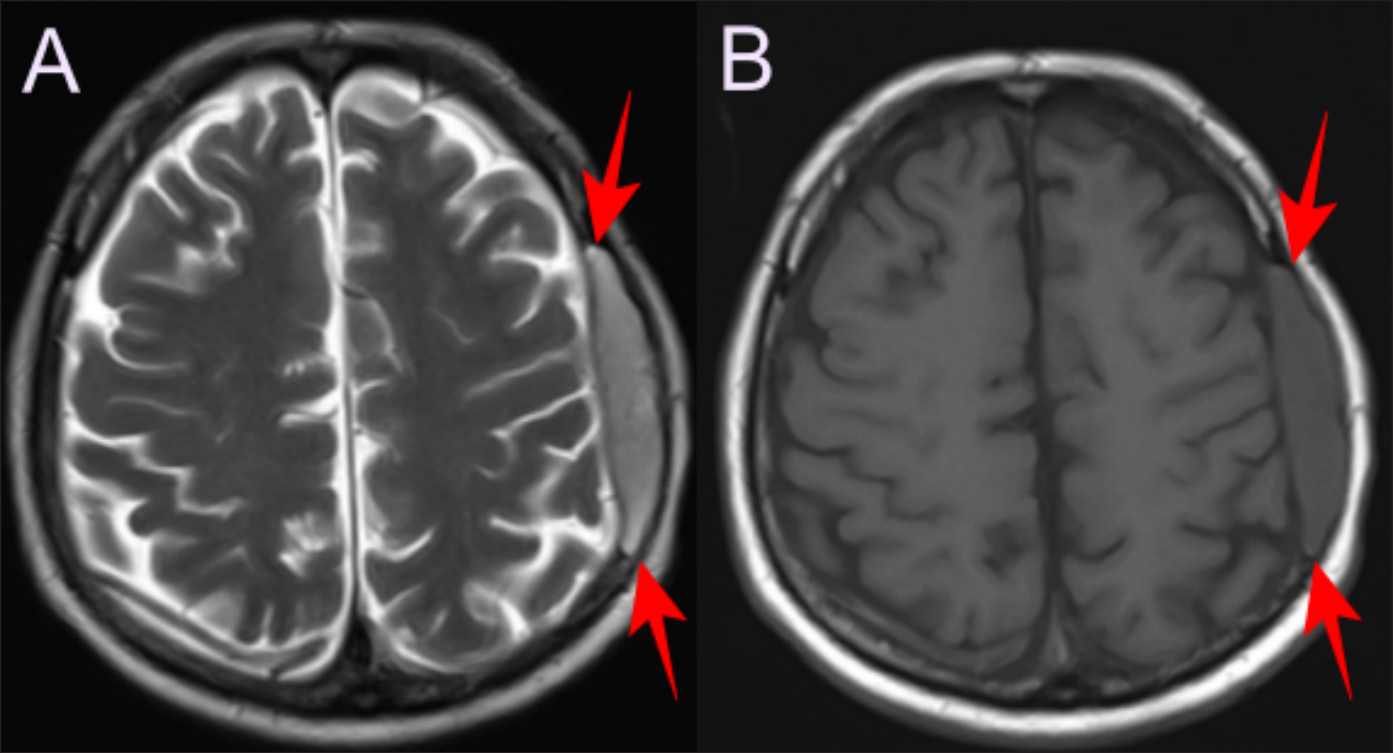


**Figure 3:**
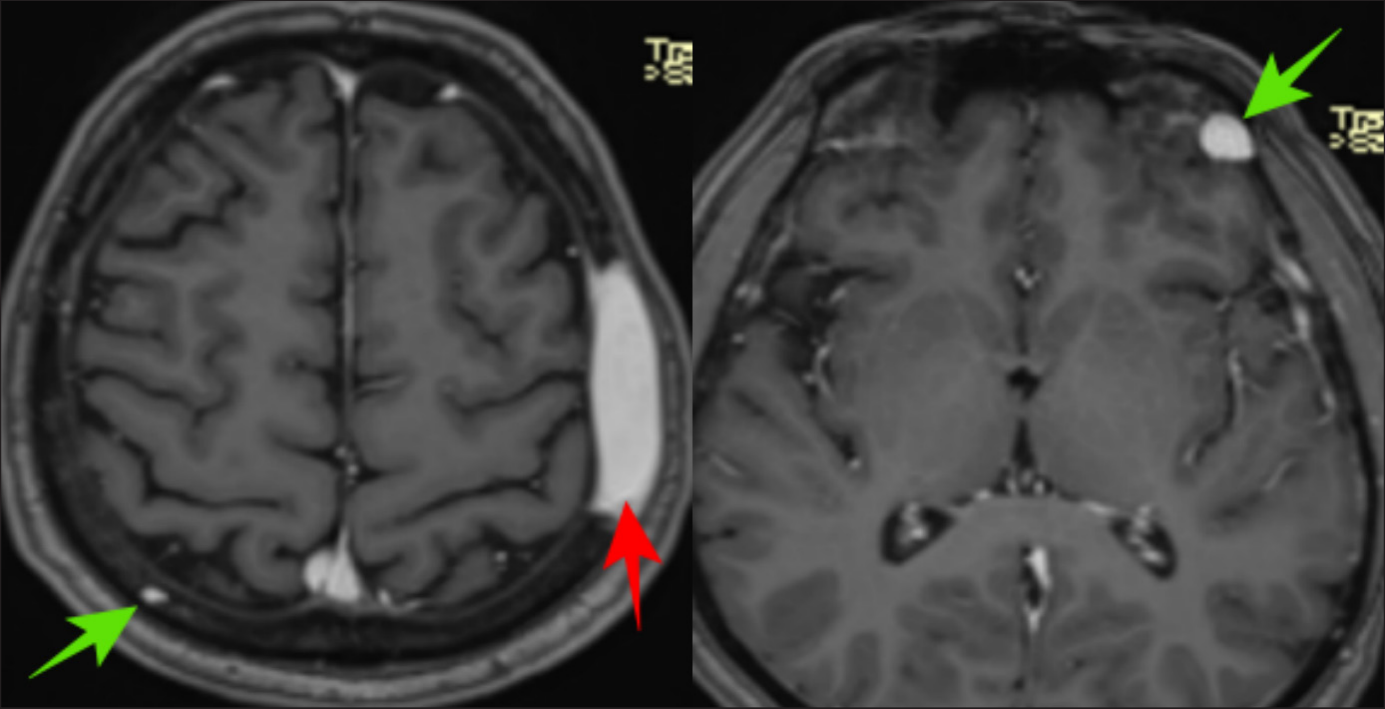


A 56-year-old male patient presented with headache and swelling on the left side of the head. His medical history was unremarkable. Hemoglobin was 11.7 g/dL. Non-contrast computed tomography (CT) of the brain showed an expansile soft tissue lesion eroding the frontoparietal bone structures extending subcutaneously from the epidural space, and compressing the brain parenchyma on the left (Figure 1). Magnetic resonance imaging (MRI) showed that the lesion was hyperintense on T2-weighted images (WI) (Figure 2A), iso- to hypointense on T1WI (Figure 2B) and homogenously enhancing on contrast imges (Figure 3; red arrow). Smaller similar lesions were also observed in the ethmoid bone, orbit and other calvarial bone structures. (Figure 3; green arrows). Immunohistochemical examination confirmed the diagnosis of extramedullary hematopoiesis (EMH) in the frontoparietal bone lesion, characterized by diffuse staining with myeloperoxidase and CD33, indicating dominance of the myeloid series. The patient underwent a whole body CT scan and no other foci were found.

## Comments

EMH acts as the body’s compensatory mechanism to meet the demand for erythropoiesis. Thalassaemia primarily causes EMH in children, while myelofibrosis is more common in adults. In addition to the bone marrow, EMH can occur in a wide variety of tissue and organss, including the liver, spleen, lymph nodes, adrenal glands, thymus, pleura, pulmonary interstitium, skin, gastrointestinal tract, paranasal sinuses, and even the dura mater. Intracranial EMH is particularly rare. Clinical evaluation with imaging findings is typically used for the diagnosis of EMH. On MRI, lesions exhibit iso-hypointense signal on T1WI and hypointense signals on T2WI due to the presence of hemosiderin. However, the lesions were slightly hyperintense on T2WI in our case. These lesions are defined as extra-axial masses that can potentially compress the underlying neuroparenchyma and resemble conditions such as metastases, angiomatous meningioma, chloroma or epidural hematoma [1].
